# Hemostasis radiotherapy for gastric cancer: Usefulness of the gastric cancer to spleen apparent diffusion coefficient ratio

**DOI:** 10.1016/j.radcr.2021.09.068

**Published:** 2021-10-28

**Authors:** Osamu Tanaka, Tomomichi Matsushita, Ryoshu Maejima, Shuji Kariya, Takuya Taniguchi, Kousei Ono, Chiyoko Makita, Masayuki Matsuo

**Affiliations:** a Department of Radiation Oncology, Asahi University Hospital, 3-23 Hashimoto-cho, Gifu City, Gifu, 500-8523, Japan; b Department of Gastroenterology, Gifu Red Cross Hospital, 3-36 Iwakura-cho, Gifu, 502-8511, Japan; c Department of Radiology, Gifu University Hospital, 1-1 Yanagido, Gifu, 501-1194, Japan

**Keywords:** Radiotherapy, Gastric cancer, Diffusion-weighted magnetic resonance imaging, Hemostatics, Endoscopy

## Abstract

The hemostatic effect of radiation therapy on gastric cancer with bleeding is known. However, blood tests and endoscopes are mainly used to determine the therapeutic effect. Additionally, magnetic resonance imaging has been reported to be useful when needed because endoscopes are invasive. In this study, magnetic resonance diffusion-weighted imaging was used to evaluate the hemostatic effect of gastric cancer. The hemostatic effect and apparent diffusion coefficient value were correlated. The apparent diffusion coefficient value was also effective in salvage irradiation during rebleeding. Although the apparent diffusion coefficient value of gastric cancer did not change during rescue irradiation, the degree of hemostatic effect could be evaluated in more detail by using the ratio of the apparent diffusion coefficient values of diffusion-weighted imaging of gastric cancer and the spleen. In the future, it would be desirable to use diffusion-weighted imaging instead of endoscopy to evaluate the gastric cancer to spleen apparent diffusion coefficient ratio in a large number of cases.

## Introduction

The hemostatic effect of radiotherapy on gastric cancer has been confirmed previously by our group and Tey *et al.* in prospective trials [1,2]. Our study reported the effectiveness of hemostatic irradiation and the possibility of re-irradiation [3,4], and Tey *et al.* reported on quality of life. We also reported the usefulness of magnetic resonance (MR) diffusion-weighted imaging (DWI) as a method for determining the effect of radiotherapy on gastric cancer other than endoscopy [5]. Other studies have reported the usefulness of hemostatic radiotherapy for gastric cancer [6], [7], [8], [9], [10], [11]. Here we report the potential for DWI in salvage re-irradiation of hemostatic radiation for gastric cancer.

## Case report

**Fig. 1:**
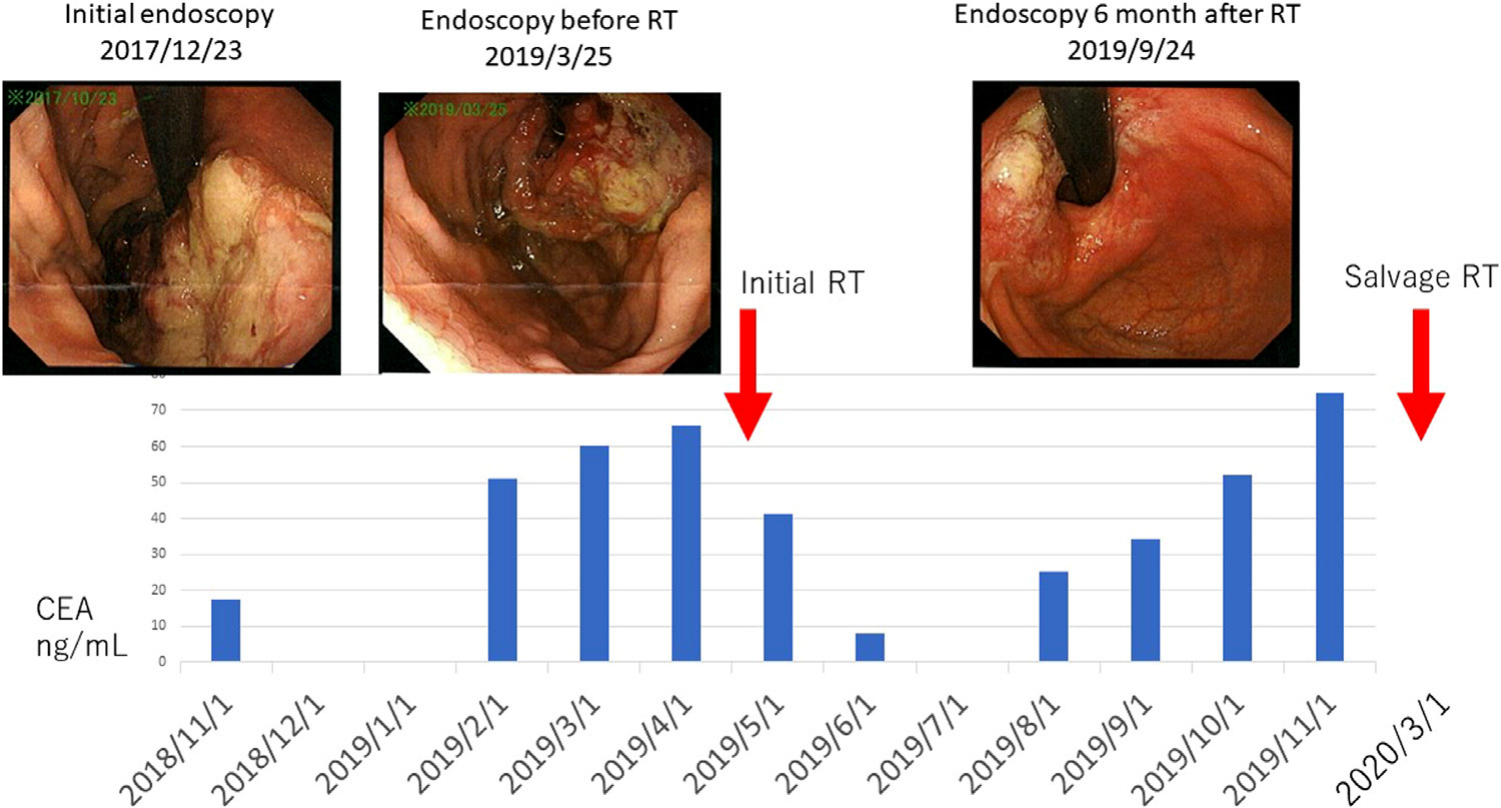
Series of treatments and changes in tumor markers. Carcinoembryonic antigen (CEA) increased with tumor growth but decreased with radiotherapy. Initial radiotherapy irradiated the entire stomach with 20 Gy/5 fractions, and salvage radiotherapy irradiated the tumor area with 15 Gy/5 fractions. Endoscopic images, CEA level (blue bar), and time of radiotherapy (red arrow) are shown (Color version of figure is available online)

A 76-year-old female was hospitalized with nausea and melena. Upper-tract endoscopy was performed, and she was diagnosed as adenocarcinoma with Borman type-3 bleeding accompanied by cardiac stenosis (Fig. 1). Liver metastasis was detected by diagnostic computed tomography (CT). She was diagnosed as stage-4 advanced gastric cancer.

We started systemic chemotherapy first because the patient was in stage 4. She received nine cycles of S-1 plus oxaliplatin (SOX) (100 mg/day S-1 for 2 weeks with 100 mg/m^2^ oxaliplatin on day 1 every 3 weeks) as first-line chemotherapy. However, about 10 months after treatment initiation, the tumor began to grow. Ramucirumab plus paclitaxel (RAM + PTX) (RAM 8 mg/kg intravenously on days 1 and 15 of a 28-day cycle plus PTX 80 mg/m^2^ intravenously on days 1, 8, and 15 of a 28-day cycle) was administered for seven cycles, but after 8 months, the tumor had grown to the extent that it was accompanied by bleeding, so a request for radiotherapy was made.

The patient was referred to our department 18 months after the first systemic chemotherapy. Her carcinoembryonic antigen (CEA) was gradually increasing (Fig. 1). Since the patient's anemia was progressing, we judged that early treatment was necessary. We tried endoscopic treatment, but it was not possible, so we performed radiotherapy, which was completed within 1 week of 20 Gy/5 fractions. The irradiation range was determined to be the entire stomach. Subsequently, she started treatment with nivolmab (240 mg/m^2^ intravenously biweekly) because she had a hemostatic effect. However, she underwent salvage hemostatic irradiation to treat her partially bleeding stomach with 15 Gy/5 fractions at 1 year after the initial RT.

We have previously reported the effectiveness of DWI for determining the effectiveness of hemostatic irradiation for gastric cancer [3], [4], [5]. DWI was performed immediately before, 1 month after, 3 months after, and 6 months after treatment. The patient received salvage hemostatic irradiation and DWI immediately before, 1 month after, and 3 months after treatment during salvage irradiation. We have previously reported efficacy in hemostatic irradiation for gastric cancer [1], but when irradiating the entire stomach, the spleen may fall within the irradiation range. However, it is known that the apparent diffusion coefficient (ADC) of the spleen shows an almost constant value (the ADC is low) regardless of whether it is inside or outside the irradiation range. To date, we have only reported changes in gastric ADC, but in the present case, we decided to measure the ADCs for gastric cancer and the spleen as well. In addition, the ratio of ADCs for the stomach and spleen was evaluated.

**Fig. 2:**
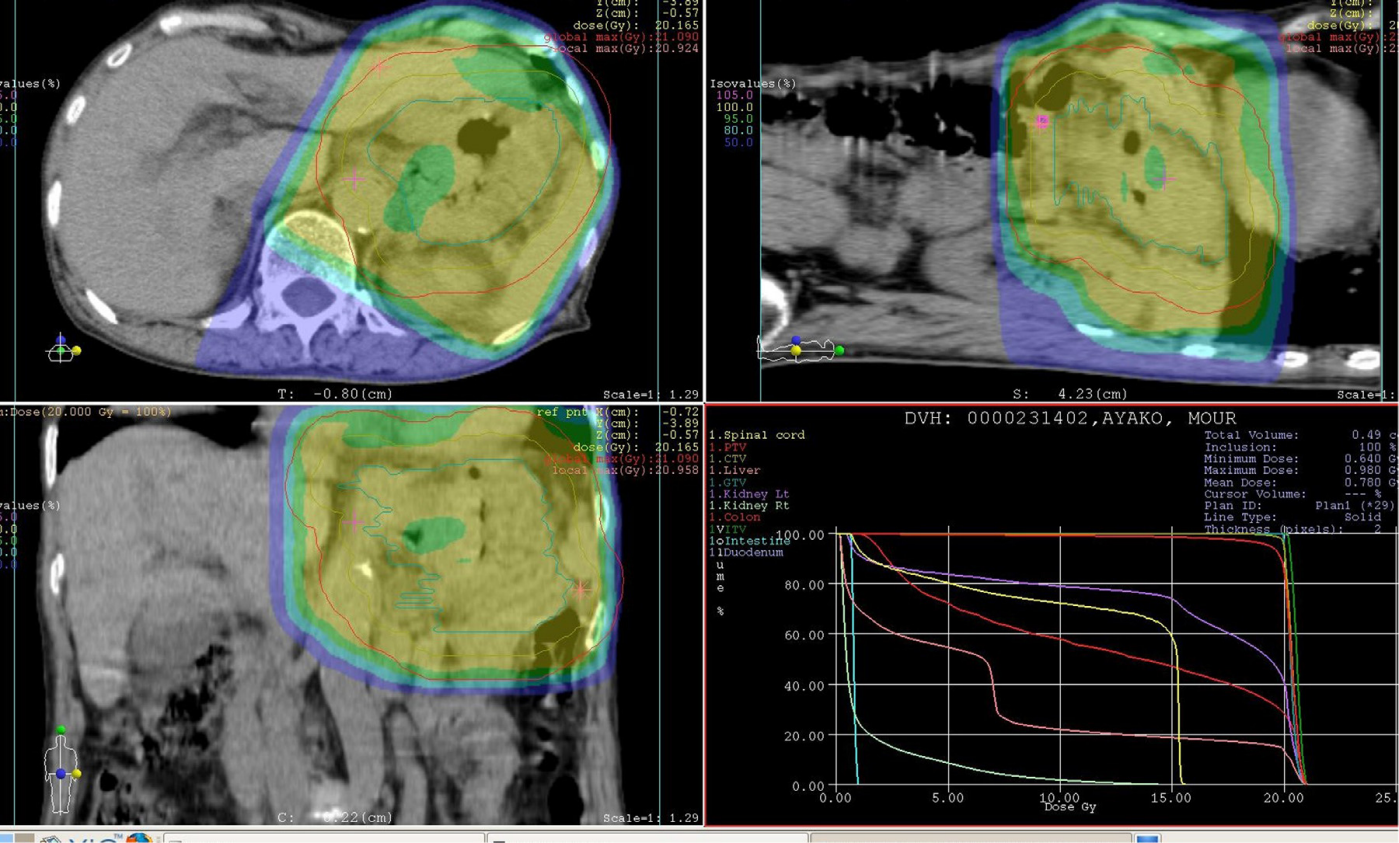
Radiotherapy planning. Contouring of the entire outer wall of the stomach. The area that is 100% irradiated at 20 Gy is shown in yellow, the area that is 95% irradiated is in green, and the area that is 50% irradiated is in blue (Color version of figure is available online)

We performed 20 Gy/5 fractions of radiotherapy (Elekta Synergy System, Elekta Ltd, Crawley, UK). Tumor spread was not correctly measurable, so we contoured the entire outer wall of the stomach. From the contour line, the clinical target volume (CTV) was contoured with a 1-cm margin from the outer stomach wall, and the planning target volume was contoured with a 1-cm margin from the CTV. The dose color wash is shown in Fig. 2.

The following imaging parameters for radiotherapy was used. Plain CT (16-row multidetector CT; Alexion, Toshiba Medical System; Otawara, Japan) and MR imaging (Achieva, Philips Medical Systems, Best, The Netherlands) were performed to investigate the extent of tumor development.

Parameters for CT: slice thickness, 2 mm; field of view, 50 cm × 50 cm; and settings of 150 mAs and 120 kV.

Parameters for T2-weighted imaging (T2-WI): fast spin-echo; repetition time (TR)/echo time (TE) in ms, 433/80; number of sample (signals) averaged (NSA), 1; matrix, 256 × 204.

Parameters for DWI: gradient echo; TR/TE in ms, 1200/65; NSA, 5; matrix, 80 × 142; B-value, 1000.

The gastric wall intensities on DWI before radiotherapy (day 0), 30 days after radiotherapy (day 30), and 90 days after radiotherapy (day 90) were compared.

**Fig. 3:**
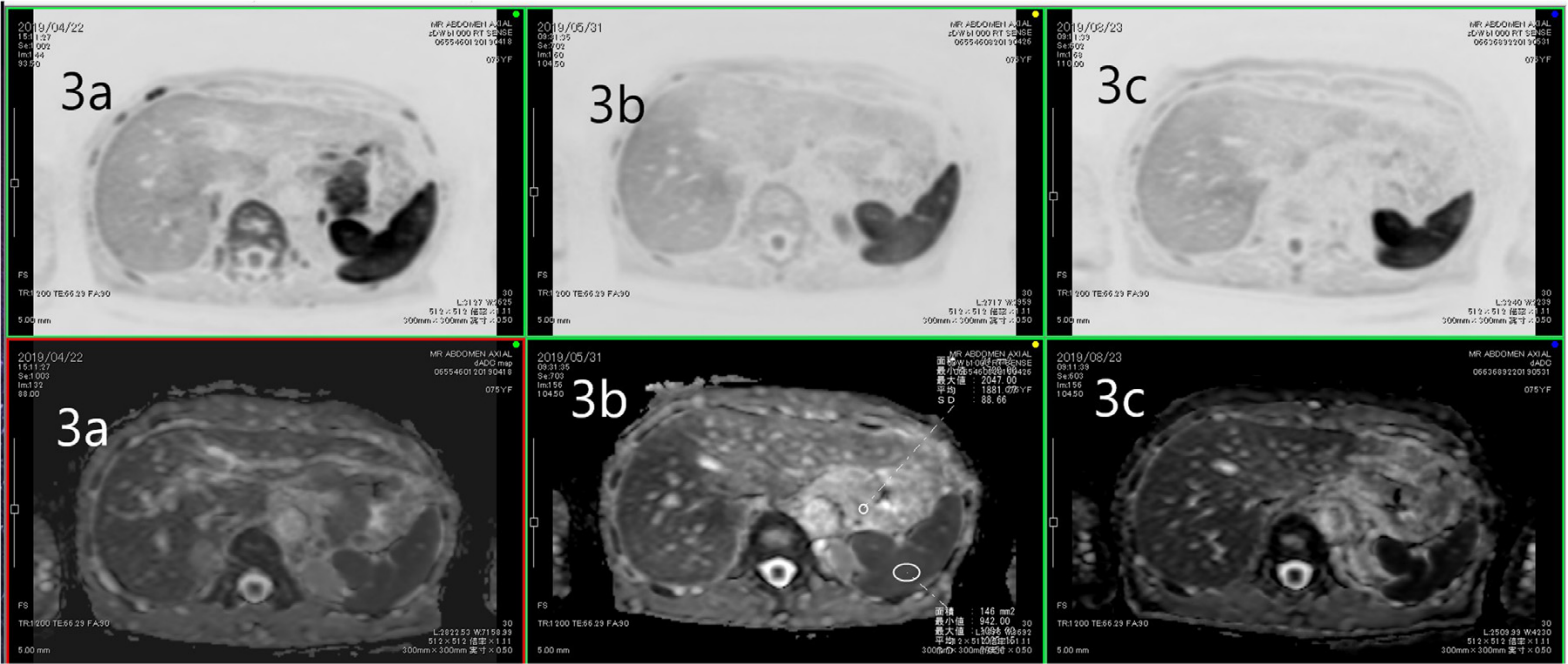
Change in the magnetic resonance (MR) diffusion-weighted imaging (DWI). (A) Day 0 of radiotherapy: High intensity of the increased gastric wall thickness is shown. No obvious change from before radiotherapy in comparison with the spleen's intensity is observed. (B) Diffusion-weighted image 2 months after the initial radiotherapy. The gastric wall signal intensity is decreased relative to that in the previous study. (C) Four months after the initial radiotherapy. The gastric wall signal intensity was the same as that in the previous study

Signal changes in DWI before and 30 days after radiotherapy in the tumor were not observed (Fig. 3A). After radiotherapy, hemoglobin stability was recognized for 1 week, which suggested a response to hemostasis irradiation. Ninety days after treatment, the DWI of the tumor showed signal decrease (Fig. 3B), and no progression of anemia was observed by the blood test. At the 6-month post-radiotherapy follow-up, endoscopic findings showed that the tumor had become ulcerated and flattened (Fig. 1). The ADC value remained high (Fig. 3C).

**Fig. 4:**
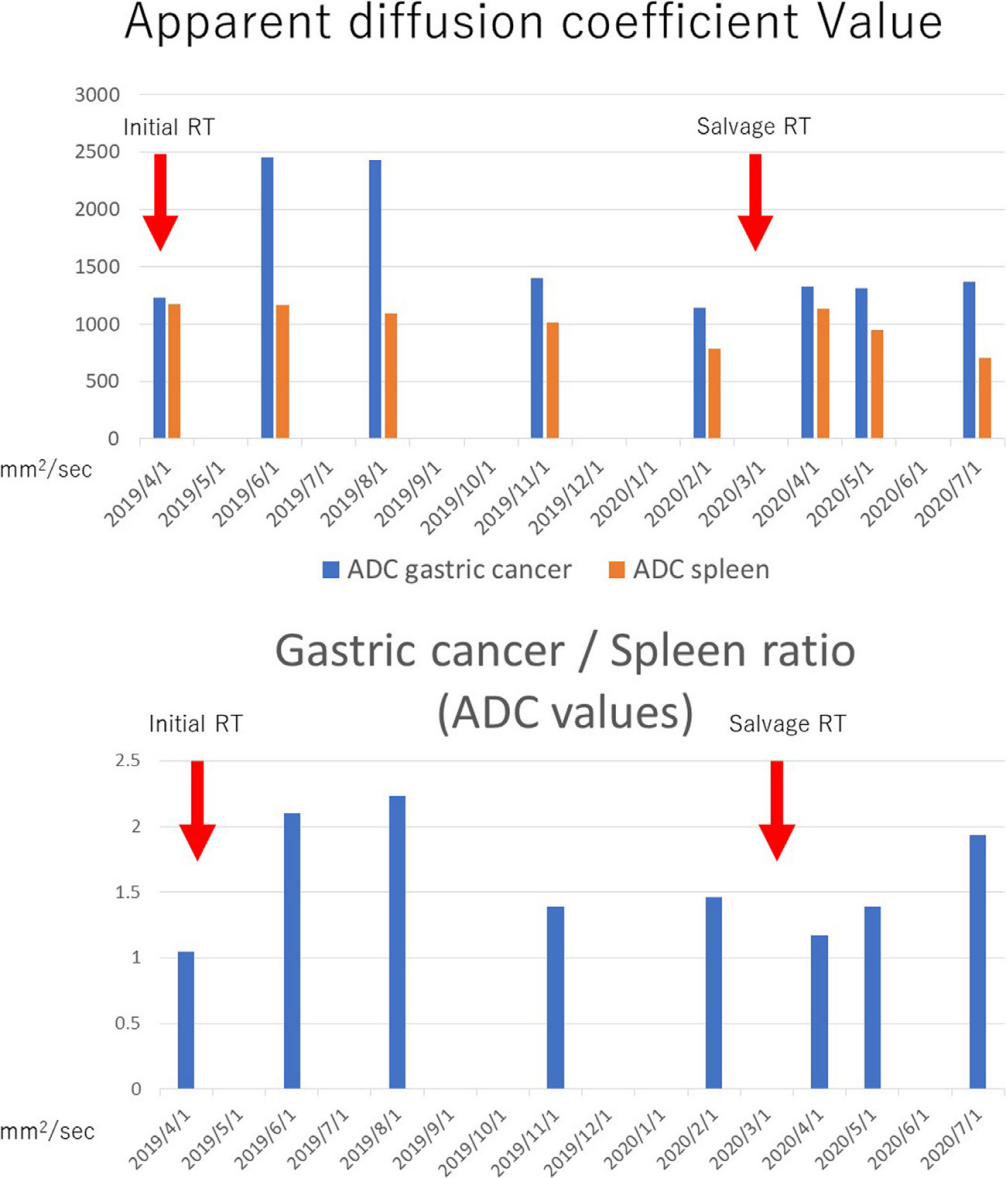
Changes in apparent diffusion coefficient (ADC) values of gastric cancer and spleen before and after radiation therapy (A) Series of ADC values of gastric cancer during radiotherapy. The change in the ADC value also increased when the hemostatic effect of gastric cancer was observed in the first radiotherapy. In the salvage irradiation, the ADC of gastric cancer did not change even after irradiation. (B) The ADC values of gastric cancer and the spleen were measured and their ratio calculated. Relief irradiation was also successful, although it was not known if success could be shown by the ADC value of gastric cancer alone, but the therapeutic effect could be determined by calculating the gastric cancer to spleen ADC ratio

Endoscopy is more invasive in patients with a short prognosis and was minimally performed. Tumor markers (CEA) and hemoglobin levels were used to measure tumor growth. When anemia was observed, we performed endoscopy. MR DWI has previously been reported to be associated with tumor shrinkage. We performed MR DWI and observed that our patient's CEA level had decreased after irradiation and increased when the tumor grew (Fig. 1). Regarding DWI, the ADC value increased after irradiation and decreased when the irradiation effect decreased (Fig. 4A). Focusing only on gastric cancer, the ADC of salvage radiotherapy did not appear to change much, but when the gastric cancer to spleen ADC ratio was calculated, the ratios of both the initial radiotherapy and salvage radiotherapy increased after the radiotherapy (Fig. 4B). Six months after the salvage irradiation, tumor re-growth was observed, and metastasis was observed in the fourth cervical spine. Radiotherapy of 8 Gy per fraction was performed for metastasis. No improvement was observed after administration of nivolumab, so chemotherapy was discontinued and home medical care was started. The patient passed away 3 years after the initial treatment.

## Discussion

Systemic chemotherapy is the first choice for patients with advanced gastric cancer who have metastases. However, bypass surgery may be necessary if there is tumor stenosis. In addition, if the tumor can be made smaller by chemotherapy, it may become operable. It is not known how surgery on patients with advanced gastric cancer contributes to survival. Therefore, the practice has been to switch to a different type of chemotherapy, but in recent years, evidence of the benefit of hemostatic irradiation for gastric cancer has been gradually increasing, although the dose and division method have not yet been determined. There is still no strong evidence of a benefit for starting chemotherapy first or local radiotherapy first.

Previously, our group and Tey *et al.* have conducted prospective trials on the effects of hemostatic irradiation for gastric cancer [2]. Our treatment method involved 20 Gy/5 fractions hemostasis irradiation for patients whose bleeding could not be stopped by endoscopic treatment. In such cases, a hemostatic effect was observed in 25/31 (80%) of patients. Subsequently, 15 Gy/5 fractions treatment was administered to patients who had rebleeding and wished to be re-irradiated. Hemostasis was observed in 6/6 (100%) [1]. On the other hand, according to the report by Tey *et al.*, the same therapeutic degree of effect was observed in 40/50 (80%) of their patients [2]. However, their method involved 36 Gy/12 fractions and took >2 weeks to complete the irradiation treatment over weekends. As a result, their study resulted in death during treatment in 6/50 (12%) patients. From this point of view, a protocol that can complete the treatment at an early stage would be desirable. We think that a fixed prospective comparative study of 20 Gy/5 fractions and 36 Gy/12 fractions would be useful in the future.

We have previously reported the efficacy and safety of re-irradiation [1,3,4]. By lowering the initial irradiation dose, the OAR dose in the case of re-irradiation could be lowered. Therefore, it was possible to perform irradiation without exceeding the regulation for surrounding organs even after re-irradiation. Regarding DWI, it has also been reported that ADC was increased by irradiating gastric cancer [5]. In our case, we performed DWI and measured ADC before and after re-irradiation. As a result, an increase in ADC was observed by re-irradiation, as in the first irradiation, but there was no significant change. The ADC of the spleen is generally low, but due to the wide irradiation range of gastric cancer, the spleen is often irradiated, so we also measured the ADC of the spleen. The spleen remains almost unchanged when exposed to radiation and its ADC always appears low. However, the ADC ratio between the stomach and the spleen was calculated. By measuring the ratio in this way, it may be possible to make a detailed imaging judgment of the effect of gastric cancer irradiation.

Regarding tumor markers, CEA levels decrease with irradiation but increase when the hemostatic effect disappears. And again, bleeding may require re-irradiation. However, endoscopic observation is painful for patients with a short prognosis. Therefore, non-invasive tests should be prioritized, and information on gastric cancer in patients can be obtained by non-invasive imaging examinations or from tumor markers. The gastric/spleen ADC ratio ​​may be a useful approach in the future, but accumulation of cases is needed to verify its usefulness.

In conclusion, we evaluated a new non-invasive method for determining the effect of hemostatic irradiation for gastric cancer. The ADC values of gastric cancer and the spleen were measured by performing DWI followed by calculating the gastric cancer/spleen ADC ratio. The ratio increased when radiotherapy was performed in the same way for both initial irradiation and salvage irradiation.

## Ethical approval

This study was approved by the Ethics Committee of Asahi University Hospital. *Competing interest*: No benefits in any form have been received or will be received from a commercial party related directly or indirectly to the subject of this article.

## Patient consent

We received written informed consent from the patient.
